# The macrophage as a neuroimmune sensor and effector: neural control of inflammation across tissues and disease

**DOI:** 10.1007/s00011-026-02401-8

**Published:** 2026-10-25

**Authors:** Renato S. da Cruz, Luana S. Lopes, Alec N. S. Pachiega, Débora S. A. Colombari, Eduardo Colombari, Daniel B. Zoccal, Pedro L. Katayama

**Affiliations:** Department of Physiology and Pathology, School of Dentistry, São Paulo State University (UNESP), Rua Humaitá 1680, Araraquara, 14801-903 São Paulo Brazil

**Keywords:** Macrophage, Microglia, Neuroimmune communication, Inflammatory reflex, Carotid body, Neuroinflammation

## Abstract

**Background:**

Once viewed mainly as scavengers of innate immunity, macrophages are now recognized as constitutive partners of the nervous system. In neural tissue their identity is instructed by neurons and glia, and in turn they shape neural function — pruning synapses, tuning neuronal excitability, and executing the autonomic commands that restrain inflammation.

**Purpose:**

Here we advance a mechanistic model built on three principles: that neural and glial signals specify macrophage identity on a shared myeloid template; that the macrophage is a bidirectional sensor–effector node within autonomic reflex arcs, both relaying neural output and generating the cytokine signals that afferent nerves detect; and that this sensor–effector arc is modular, recurring across brain, spleen, gut, heart, and adipose tissue. We give particular attention to the afferent limb of the inflammatory reflex, which now extends beyond the vagus to nociceptors and to the carotid body, an arterial chemoreceptor that senses circulating inflammatory signals and recruits a sympathetic anti-inflammatory pathway, and to the stress-responsive circuitry through which sympathetic outflow drives myelopoiesis, neuroinflammation, and sickness behavior. We then turn to disease, in which macrophage function is altered by a diseased or aged niche in neurodegeneration, chronic pain, gut–brain disorders, and metabolic disease.

**Conclusions:**

Whether the neuroimmune circuits described here are themselves disrupted in these conditions, rather than merely coexisting with them, is largely untested, and we outline what such evidence would require. We close by arguing that tuning these circuits, rather than broadly suppressing macrophage activity, is the more plausible therapeutic goal.

## Introduction: reuniting two systems around one cell

For much of the twentieth century, neuroscience and immunology developed as separate disciplines, in part because of the concept of central nervous system (CNS) immune privilege and an assumption that neural and immune signaling used largely non-overlapping mechanisms. That view no longer holds: the two systems communicate continuously through shared mediators, receptors, and anatomical interfaces, and together regulate homeostasis and the response to injury and infection [1, 2]. The macrophage contributes to this integration at multiple sites. As the most broadly distributed innate immune cell, present in every organ including the brain, it receives signals from the nervous system and, in turn, influences neuronal function through the mediators it secretes [3, 4].

Several lines of evidence support treating the macrophage as a neuroimmune cell — by which we mean a niche-instructed functional state, prominent in nerve-associated, visceral, and CNS-resident populations, rather than an intrinsic property of every tissue macrophage. Microglia, the resident macrophages of the brain parenchyma, are not only immune sentinels but contribute to neural circuit formation by removing synapses during development and by supporting neurogenesis and learning in adult life [5–7]. In the periphery, macrophages lie in close physical association with sympathetic, sensory, and enteric neurons, where they clear neurotransmitters, regulate organ function, and modulate neuronal activity [8–10]. The afferent and efferent arms of the autonomic nervous system use macrophages as both sensors and effectors of systemic inflammation [11–13], and the macrophage-nociceptor interface contributes to the generation, amplification, and resolution of pain [14–16].

This review is organized around the neuroimmune identity of the macrophage — the niche-instructed phenotypic state that neural and glial cues impose on macrophages resident in and around nervous tissue, not a reassignment of the lineage as a whole — rather than its general immunological functions, and advances a single argument rather than an encyclopedic catalog. We organize the field around three principles that together constitute a mechanistic model. First, macrophage identity in and around the nervous system is not intrinsic but instructed: neurons and glia supply the signals — TGF-β, CX3CL1, IL-34, and CSF1 — that impose tissue-specific programs on a common myeloid template. Second, the macrophage is a bidirectional node in autonomic reflex arcs, simultaneously an effector that executes neural commands and a sensor whose cytokine output is read by afferent nerves, rather than a passive target of neural tone. Third, this sensor-effector arc is modular and recurs across tissues — brain parenchyma, spleen, gut, heart, and adipose depot — so that disease in each compartment can be read as a perturbation of one underlying circuit. Our recent identification of an arterial chemoreceptor afferent that reports circulating inflammation to the brainstem [13] illustrates the second principle sharply, and is a thread we return to throughout.

## Origins of a neuroimmune cell

The macrophage entered science through comparative biology: in Messina in 1882–1883, Elie Metchnikoff observed mobile mesenchymal cells surrounding a rose thorn inserted into a starfish larva, the founding observation of phagocytosis, and already anticipated functions beyond host defense in the scavenging of senescent host cells [4, 17]. The neuroimmune lineage has an equally deep root: between 1919 and 1921, Pio del Rio-Hortega identified microglia as a distinct CNS population and correctly inferred their mesodermal origin, phagocytic capacity, and transformation into reactive forms in disease [18]. The reunion of the macrophage lineage — peripheral macrophages together with their central-nervous-system counterparts, the microglia — with immunology followed three developments: the demonstration that the autonomic nervous system reflexively controls innate immune cytokine production [1, 12]; the discovery that microglia are yolk-sac-derived macrophages maintained independently of the bone marrow [19]; and the recognition that sensory neurons and macrophages communicate bidirectionally at sites of injury [14].

## Macrophage populations of the nervous system

**Fig. 1:**
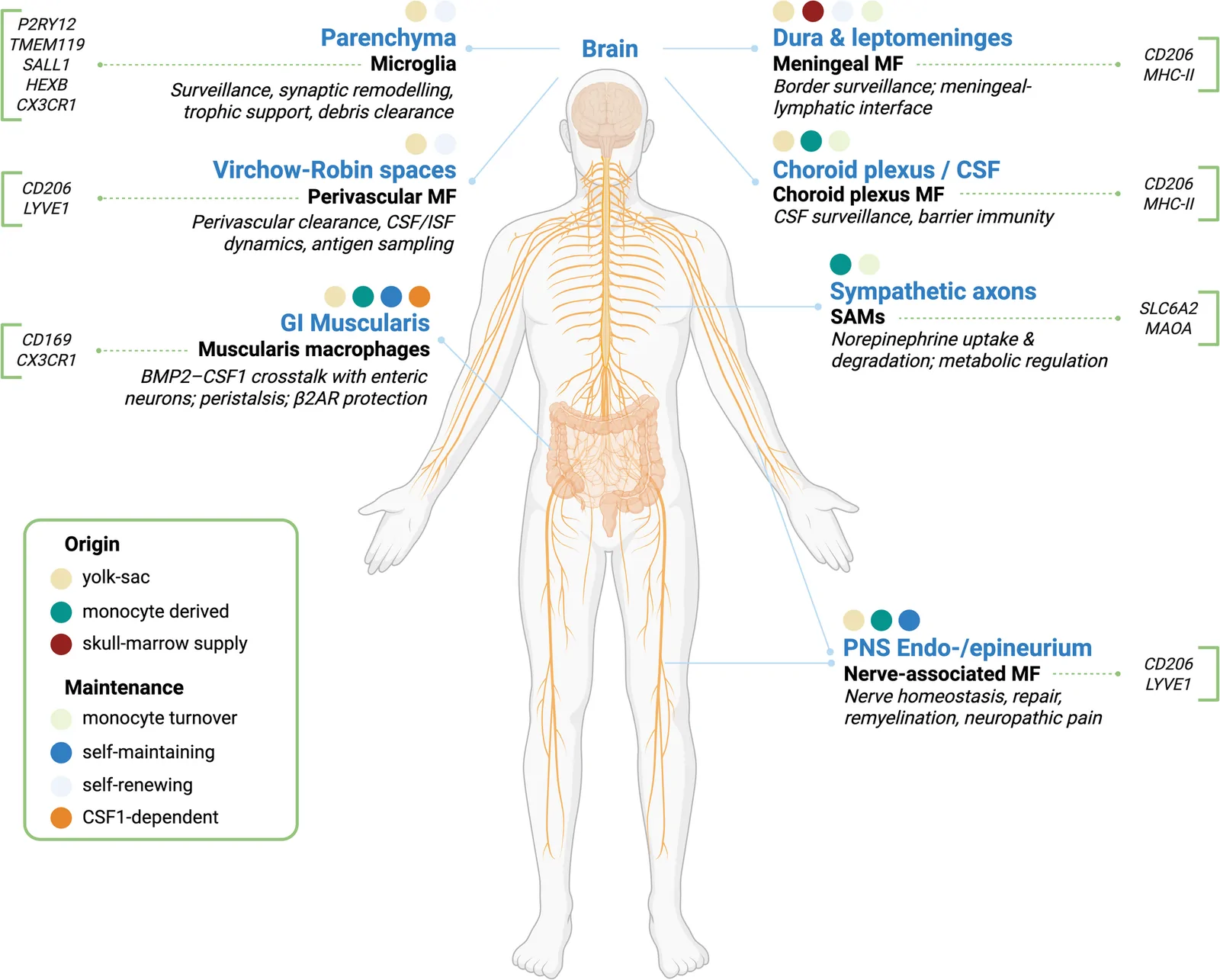
Macrophage populations of the nervous system and its interfaces. Resident macrophages populate every compartment of the nervous system and are specified by the niche they occupy. In the central nervous system, parenchymal microglia (signature genes P2RY12, TMEM119, SALL1, HEXB, CX3CR1) perform surveillance, synaptic remodeling, trophic support and debris clearance, while border-associated macrophages occupy the perivascular Virchow–Robin spaces (CD206, LYVE1), the dura and leptomeninges (CD206, MHC-II) and the choroid plexus and cerebrospinal fluid interface (CD206, MHC-II). In the periphery, sympathetic neuron-associated macrophages (SAMs; SLC6A2, MAOA) take up and degrade norepinephrine and thereby regulate metabolism; gastrointestinal muscularis macrophages (CD169, CX3CR1) engage in BMP2–CSF1 crosstalk with enteric neurons to support peristalsis and are protected by β2-adrenergic signaling; and nerve-associated macrophages of the endoneurium and epineurium (CD206, LYVE1) maintain nerve homeostasis, repair and remyelination and contribute to neuropathic pain. Please note that the relationships shown are based largely on rodent studies; human data confirm the parenchymal microglial signature genes and their dependence on the brain environment, and the border-associated compartments and their CD206/MRC1 and LYVE1 markers (Sect.  12). The sympathetic neuron-associated and muscularis populations, the nerve-repair functions, and the origin and maintenance assignments below are rodent-derived. The key at lower left indicates developmental origin (yolk-sac; monocyte-derived; skull-marrow supply) and maintenance mode (monocyte turnover; self-maintaining; self-renewing; CSF1-dependent). Created with BioRender.com

The CNS harbors two classes of resident macrophages: parenchymal microglia and the border-associated macrophages of the CNS interfaces (Fig. 1). Microglia constitute some 5–10% of CNS cells and tile the brain and spinal cord in a near-regular, non-overlapping array, extending highly motile processes that continuously survey the parenchyma and contact neurons, synapses, and blood vessels [20, 21]. They are defined transcriptionally by homeostatic genes — P2RY12, TMEM119, SALL1, HEXB, OLFML3 — that distinguish them from other CNS macrophages and from peripheral myeloid cells and that depend on continuous instruction by the CNS microenvironment [22–24]. Unlike most peripheral macrophages they are long-lived and self-renew locally throughout adult life, with minimal monocyte contribution under homeostasis [19].

Beyond the parenchyma, border-associated macrophages (BAMs) occupy the perivascular spaces, the meninges, and the choroid plexus. They are transcriptionally and functionally separable from microglia, expressing CD206 (MRC1), LYVE1, and MHC class II in subset-specific patterns [25–27] — a separation now confirmed with lineage-restricted genetic tools that distinguish embryo-derived brain macrophages from microglia [28]. Perivascular macrophages regulate cerebrospinal and interstitial fluid dynamics and sample antigen at the blood-brain interface; meningeal macrophages populate the dural and leptomeningeal layers; and choroid plexus macrophages survey the cerebrospinal fluid-producing epithelium [29]. Unlike microglia, some BAM populations are partially replenished by bone marrow-derived monocytes over the lifespan [25, 27]. Dural macrophages in particular are supplied by monocytes from the adjacent skull and vertebral marrow, reaching the meninges through direct osseous channels rather than the circulation [30, 31]; this marrow carries a molecular signature distinct from that of other bones and is altered in human neurological disease [32].

Macrophages are also present throughout the peripheral nervous system. Peripheral nerves harbor resident macrophages in the endoneurium and epineurium, apposed to axons and Schwann cells; they express CD206 and LYVE1, are largely self-maintaining, and carry a program instructed by the nerve microenvironment [33]. Under homeostasis they perform surveillance and debris clearance; after injury they proliferate locally and adopt programs that either support axon regeneration and remyelination or, when dysregulated, sustain neuropathic pain [33, 34]. A specialized population associates with sympathetic axons, particularly in adipose tissue: these sympathetic neuron-associated macrophages (SAMs) express the norepinephrine transporter SLC6A2 and monoamine oxidase A, enabling them to import and catabolize norepinephrine released from sympathetic terminals [10]. By clearing the neurotransmitter they set its availability to target cells and thereby influence lipolysis and thermogenesis; in obesity their numbers increase and their enhanced clearance blunts sympathetic drive to fat [10].

The gastrointestinal tract contains the largest pool of macrophages in the body, and of particular neuroimmune importance are the muscularis macrophages lying within the smooth muscle layers alongside the enteric nervous system. Muller et al. [8] demonstrated a bidirectional trophic dialogue between them: macrophage-derived bone morphogenetic protein 2 (BMP2) acts on BMP receptor-expressing enteric neurons to regulate motility, while the neurons reciprocally produce colony-stimulating factor 1 (CSF1) required for macrophage maintenance. Gabanyi et al. [9] showed that luminal infection drives muscularis macrophages toward a tissue-protective phenotype through β2-adrenergic signaling in response to sympathetic norepinephrine, and De Schepper et al. [35] established that enteric macrophages are self-maintaining and provide neurotrophic support essential for enteric neuron survival. The two cell types are therefore mutually dependent — one of the clearest examples of a peripheral macrophage-neuron unit.

## Neural instruction of macrophage identity

The developmental origin of microglia is the foundation of their neuroimmune identity. Using inducible fate-mapping, Ginhoux et al. [19] demonstrated that adult microglia derive almost exclusively from primitive macrophages generated in the embryonic yolk sac, which colonize the developing neuroepithelium before the establishment of definitive hematopoiesis and before closure of the blood-brain barrier. Colonization depends on the transcription factors PU.1 and IRF8 and on CSF1R signaling, and once seeded the microglial pool is maintained by local self-renewal essentially independent of the bone marrow throughout life [36, 37]. This embryonic origin and lifelong residence mean that microglia age with the brain they inhabit, accumulating cell-intrinsic changes with direct consequences for neurodegeneration.

Although microglia share a common myeloid origin with peripheral macrophages, their identity is imposed by the CNS microenvironment — an instance of the principle that every resident-macrophage population is instructed by its niche [38]. TGF-β from the neural environment is required for the homeostatic microglial signature: Butovsky et al. [23] showed that it drives the microglia-specific genes distinguishing these cells from all other macrophages, and that loss of TGF-β signaling collapses microglial identity. Neuronal fractalkine (CX3CL1), acting through CX3CR1, constrains microglial activation and is essential for normal synaptic pruning [5]; neuronal CD200 engages CD200R to deliver a tonic restraining signal [39]; and IL-34 and CSF1 act through CSF1R to sustain microglial survival [36, 40]. Identity is not fixed: transplantation and depletion-repopulation experiments show that the homeostatic signature requires continuous environmental instruction and is lost when cells leave the CNS [23]. This signature is best defined in mouse; human microglia follow the same broad logic but differ in aspects of their transcriptional profile, so murine identity programs should be extrapolated to human disease with care [24].

To perform these functions microglia express a specialized repertoire of sensing receptors. Hickman et al. [22] defined the microglial sensome — the transcripts encoding receptors through which microglia perceive their environment, including purinergic, chemokine, and pattern recognition receptors and the TREM2-DAP12 module. This apparatus underlies the constant surveillance seen by two-photon imaging, in which microglial processes sample the parenchyma over hours and converge rapidly on injury through purinergic chemotaxis toward ATP released from damaged tissue [20, 21].

Microglial maturation also depends on the intestinal microbiota. Erny et al. [41] showed that germ-free mice have defective microglia with altered morphology and impaired innate immune responses, and that the defect is corrected by recolonization or by short-chain fatty acids. The functional state of brain macrophages is therefore shaped not only by local neural signals but also by signals originating in the intestine, connecting the microglial compartment to the gut-brain axis discussed in Sect. 10.

## Homeostatic neuroimmune functions

Microglia eliminate synapses and thereby refine neural circuits. Stevens et al. [42] showed that the classical complement cascade marks synapses for elimination, C1q localizing to subsets of synapses and, through C3 deposition, tagging them for removal; Schafer et al. [6] showed that microglia engulf complement-tagged presynaptic inputs in an activity- and complement receptor 3-dependent manner, with less active inputs preferentially eliminated; and Paolicelli et al. [5] showed that this engulfment requires neuronal CX3CL1 signaling to microglial CX3CR1. Pruning is therefore a process in which an immune cell uses immune molecules to perform a developmental function. The model is influential but not uncontested: astrocytes eliminate synapses in parallel through MEGF10 and MERTK, and whether complement-dependent microglial pruning is causal rather than correlative in human disease remains debated [43].

Microglia support neural function well beyond pruning. They provide direct trophic support: Parkhurst et al. [7] showed that microglia-derived brain-derived neurotrophic factor (BDNF) promotes learning-dependent synapse formation, and that removing microglial BDNF impairs motor learning. Microglia thereby contribute to synaptic plasticity, circuit maintenance, and cognition in addition to host defense.

In the periphery, the homeostatic neuroimmune role of macrophages is exemplified by the control of organ function. Macrophage-derived BMP2 modulates the enteric neurons that govern peristalsis, so that macrophage signals tune the rhythm of gastrointestinal contraction, while sympathetic β2-adrenergic input drives a tissue-protective macrophage program during enteric infection [8, 9]; and sympathetic neuron-associated macrophages, by clearing norepinephrine locally, tune its availability to target cells and thereby shape lipolysis and thermogenesis (Sect. 3) [10]. Peripheral macrophages are accordingly active regulators of autonomic organ function rather than passive immune sentinels.

## The molecular dialogue at the neuroimmune interface

**Fig. 2:**
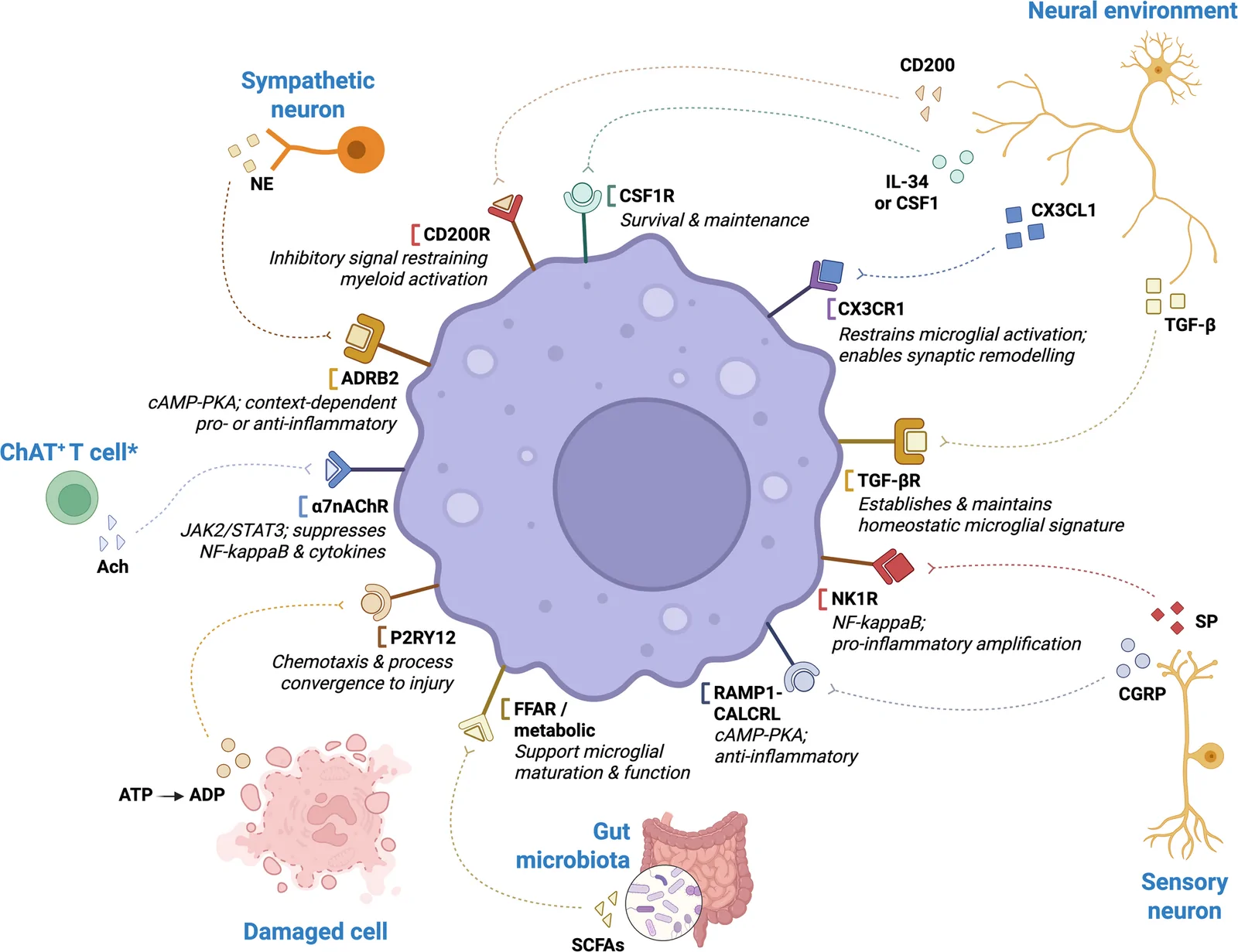
The molecular dialogue at the neuroimmune interface: inputs to the macrophage. The macrophage integrates signals from neurons, glia, damaged cells and the gut microbiota. Instructive signals from the neural environment — TGF-β acting on TGF-βR, CX3CL1 on CX3CR1, IL-34 or CSF1 on CSF1R, and CD200 on CD200R — establish and maintain the homeostatic microglial signature, restrain myeloid activation and support survival. Autonomic and sensory neurotransmitters tune the activation state: norepinephrine from sympathetic terminals acts on ADRB2 (cAMP–PKA; context-dependent pro- or anti-inflammatory), acetylcholine on α7nAChR (JAK2/STAT3; suppression of NF-κB and cytokines), substance P on NK1R (NF-κB; pro-inflammatory amplification) and CGRP on the RAMP1–CALCRL receptor (cAMP–PKA; anti-inflammatory). ATP released from damaged cells engages P2RY12 to drive chemotaxis and process convergence to injury, and microbiota-derived short-chain fatty acids act through FFAR and metabolic receptors to support microglial maturation and function. The asterisk denotes that acetylcholine may be supplied by choline acetyltransferase-positive T cells rather than by direct cholinergic innervation (see Sect.  7.2). Created with BioRender.com

Peripheral neurons release neuropeptides and neurotransmitters that directly modulate macrophage function (Fig. 2). Substance P signals through the neurokinin-1 receptor to activate NF-κB, amplify TNF-α, IL-1β, and IL-6, and potentiate Toll-like receptor responses — a feed-forward arm of neurogenic inflammation [14, 44]. Calcitonin gene-related peptide (CGRP), by contrast, elevates macrophage cAMP through the RAMP1-coupled receptor CALCRL, activates protein kinase A, and suppresses NF-κB, exerting a net anti-inflammatory effect; vasoactive intestinal peptide acts similarly through VPAC receptors [14, 44]. The physiological reach of this control is established in vivo: CGRP-expressing sensory neurons direct neutrophil and macrophage behavior to promote tissue healing [45], and sensory neurons maintain immune homeostasis in barrier tissue through the same axis [46], so the peptidergic tone of a tissue shapes the macrophages within it. Acetylcholine and norepinephrine suppress macrophage cytokine production through α7 nicotinic and β2-adrenergic receptors respectively (Sect. 7). Macrophage inflammatory output is therefore set in part by the neurochemical composition of the local environment.

**Fig. 3:**
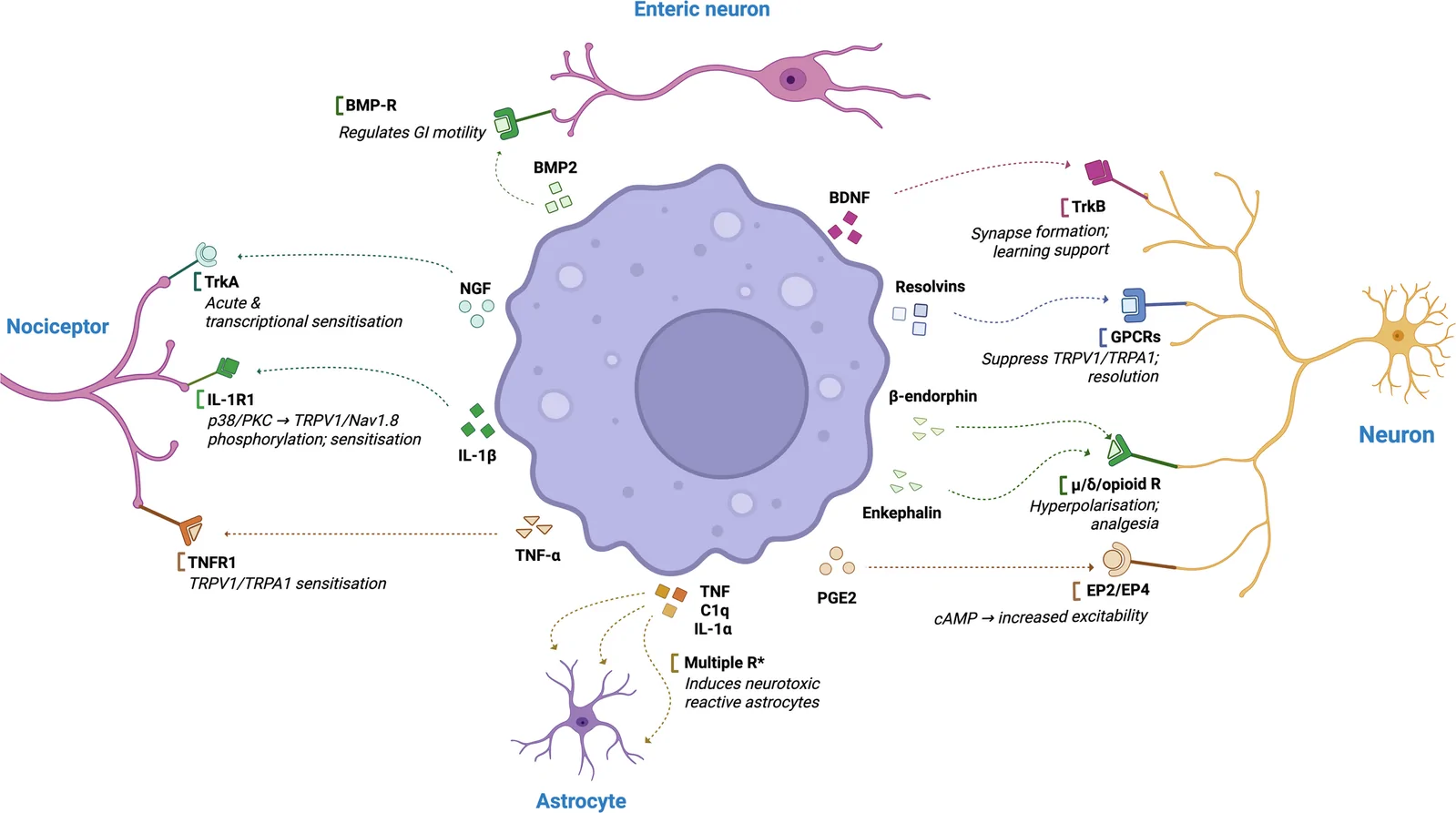
The macrophage as a source of neuroactive mediators: outputs to neurons and glia. Complementing the afferent inputs of Fig. 2, macrophages secrete mediators that reset the excitability and behavior of neurons and glia. Pro-nociceptive outputs sensitize nociceptors: NGF acts on TrkA (acute and transcriptional sensitization), IL-1β on IL-1R1 (p38/PKC → TRPV1/Nav1.8 phosphorylation) and TNF-α on TNFR1 (TRPV1/TRPA1 sensitization). Pro-resolving and trophic outputs act in the opposite direction: β-endorphin and enkephalin engage µ/δ-opioid receptors to hyperpolarize nociceptors and produce analgesia; resolvins act on neuronal GPCRs to suppress TRPV1/TRPA1 and drive resolution; BDNF acts on TrkB to support synapse formation and learning; and PGE2 acts on EP2/EP4 to increase excitability. BMP2 acts on enteric neuronal BMP receptors to regulate gastrointestinal motility. Acting on glia, macrophage- and microglia-derived TNF, C1q and IL-1α together induce neurotoxic reactive astrocytes (asterisk: multiple receptors). Created with BioRender.com

Signaling also occurs in the opposite direction (Fig. 3). Activated macrophages secrete mediators that lower the activation threshold of sensory neurons, producing the peripheral sensitization that underlies inflammatory pain. Interleukin-1beta signals through IL-1R1 on nociceptor terminals, activating p38 MAPK and PKC, which phosphorylate TRPV1 and the sodium channel Nav1.8 [44]; TNF-α sensitizes TRPV1 and TRPA1 through TNFR1; nerve growth factor acts through TrkA to produce acute sensitization and longer-lasting transcriptional changes; and prostaglandin E2 acts through EP2/EP4 receptors to increase excitability [14, 44]. Sustained nociceptor input induces central sensitization, first characterized by Woolf [47], and spinal dorsal-horn microglia are themselves major contributors to this central facilitation, so the macrophage lineage operates on both limbs of the pain pathway [44]. We use the shorthand of pro-inflammatory and pro-resolving (‘M1-like’ and ‘M2-like’) states for convenience, but the binary scheme derives largely from in vitro polarization with single stimuli; in tissue, macrophages occupy a continuous, multidimensional activation space, and consensus recommendations now favor describing activation by the specific stimulus and the markers measured [48, 49]. The point is concrete here: the same cell can simultaneously sensitize nociceptors and generate pro-resolving mediators.

Single-cell and spatial transcriptomics have allowed the macrophage-nociceptor relationship to be mapped systematically. Jain et al. [16] applied single-cell RNA sequencing to three mouse models of inflammatory pain — zymosan injection, surgical incision, and ultraviolet burn — across the full time course from onset to resolution, reconstructing injury-specific neuroimmune interactomes from co-expressed ligand-receptor pairs. Macrophage transcriptional states tracked the kinetics of pain hypersensitivity, and each injury type generated a distinct interactome, demonstrating that the macrophage-nociceptor interface is shaped by the nature of the insult rather than following a single stereotyped program [16].

Macrophages are not only generators of pain but active agents of its resolution. In a reparative state they produce opioid peptides — β-endorphin, met-enkephalin, and dynorphin — that bind µ-, δ-, and κ-opioid receptors on nociceptor terminals, hyperpolarizing the membrane and reducing firing [15]. During resolution they generate specialized pro-resolving mediators — resolvins, protectins, and maresins — that act on neuronal G-protein-coupled receptors to suppress TRPV1 and TRPA1 sensitization and inhibit dorsal root ganglion excitability [15, 50]. At sites of nerve injury, pro-regenerative macrophages supply BDNF, GDNF, and VEGF supporting axonal regrowth; conversely, when macrophage infiltration becomes chronic — as after peripheral nerve injury, where CX3CR1-dependent local proliferation of nerve-associated macrophages sustains hypersensitivity [34] — it maintains the neuropathic pain of diabetic and chemotherapy-induced neuropathy [15, 33]. The therapeutic implication, that shifting macrophage polarization toward resolution could treat pain, is taken up in Sect. 11.

## Autonomic reflex circuits

### The efferent arm: sympathetic restraint of macrophage cytokine output

Reflex control of macrophage cytokine production by the autonomic nervous system is mediated by the splanchnic sympathetic nerves. Martelli et al. [12] showed that the reflex limiting the plasma TNF-α response to endotoxin is abolished by section of the greater splanchnic sympathetic nerves but is unaffected by vagotomy, indicating that the efferent arm of the inflammatory reflex is sympathetic rather than parasympathetic. Anatomical tracing by Bratton et al. [51] found no neural connection from the vagus to the splenic sympathetic neurons, which argues against a direct vagal route to the spleen. On this basis Martelli and colleagues proposed the splanchnic anti-inflammatory pathway as the efferent arm of the reflex, in which sympathetic fibers carried in the splanchnic nerves restrain the cytokine output of macrophages in the spleen and other abdominal viscera [52, 53].

The molecular basis of this control is well characterized. Sympathetic terminals densely innervate lymphoid organs, bone marrow, liver, adipose tissue, and gut, releasing norepinephrine onto macrophage adrenergic receptors [54, 55]. Beta2-adrenergic engagement elevates cAMP, activates protein kinase A, inhibits IκB kinase, and thereby suppresses NF-κB-dependent transcription of TNF-α, IL-6, and IL-12 while promoting IL-10 [54]. Macrophages additionally express the α7 nicotinic acetylcholine receptor, through which acetylcholine suppresses NF-κB signaling and the production of TNF-α, IL-1β, IL-6, and HMGB1 [11]. SAMs additionally regulate local norepinephrine availability by uptake and degradation (Sect. 3) [10]. These catecholaminergic inputs make the macrophage a direct effector of sympathetic tone.

### A contested efferent limb: cholinergic versus splanchnic-sympathetic models

The identity of the efferent arm is, however, genuinely contested, and the dispute bears directly on how the reflex should be engaged therapeutically. In the cholinergic anti-inflammatory pathway proposed by Tracey and colleagues, efferent vagal activity ultimately delivers acetylcholine to the α7 nicotinic receptor of splenic macrophages: electrical vagal stimulation attenuates endotoxin-induced TNF-α and shock [56], the α7 receptor is required for cholinergic restraint of cytokine release [11], and the apparent absence of direct cholinergic innervation of the spleen was reconciled by a relay in which noradrenergic splenic-nerve signals engage choline acetyltransferase-positive, acetylcholine-synthesizing CD4 + T cells that furnish the acetylcholine acting on macrophage α7 receptors [57]. The splanchnic model rests on the denervation and tracing evidence set out above [12, 51], placing the efferent outflow in the sympathetic rather than the parasympathetic division.

The two models agree on the effector chemistry — both converge on suppression of NF-κB-dependent cytokine production at the macrophage — and disagree on the wiring. The open questions are whether a functional vagus-to-spleen circuit exists, whether direct or relayed; whether the choline acetyltransferase-positive T-cell relay is necessary; and how α7- and beta2-mediated signaling are weighted in vivo. The distinction is clinically consequential, since it determines whether the reflex is best engaged by vagus nerve stimulation or by targeting sympathetic and splanchnic outflow, and it cautions against reading the clinical benefit of vagus nerve stimulation as proof of a direct vagal-cholinergic mechanism [1, 55]. We adopt the splanchnic-sympathetic account here, while treating the question as open.

### The afferent arm: how the nervous system detects inflammation

A reflex requires an afferent limb, and the nervous system detects inflammation through several routes. Vagal sensory afferents express receptors for inflammatory cytokines and relay peripheral immune status to the brainstem, the pathway central to the original conception of the inflammatory reflex [1, 44]; single-cell and functional mapping of the vagal interoceptive system has begun to resolve how such visceral signals are encoded at the level of individual afferent populations [58]. Nociceptor sensory neurons also act as afferent sensors, detecting bacterial products and inflammatory mediators directly and conveying this information centrally in addition to their local actions [14, 44].

To these established routes, work from our group [13] added an arterial chemoreceptor: we identified the carotid body, the paired chemoreceptor at the carotid bifurcation classically regarded as the primary sensor of arterial oxygen, as an afferent immunological sensor. Because this limb emerged largely from our own laboratory, we flag it here as the principal point at which the present review takes a perspective, and we indicate our own contributions explicitly throughout. The carotid body expresses TNFR1, and systemic TNF-α increases its afferent discharge in the carotid sinus nerve, activating glutamatergic neurons in the nucleus tractus solitarius (NTS) that project to the rostral ventrolateral medulla (RVLM), the brainstem source of pre-sympathetic drive. This NTS-RVLM circuit raises splanchnic sympathetic nerve activity to the spleen and abdominal macrophage pools, and carotid body ablation blunted both the activation of RVLM-projecting NTS neurons and the rise in splanchnic sympathetic activity, while plasma and splenic cytokine levels after TNF-α were higher in animals subjected to carotid body ablation or splanchnic denervation [13]. Independent work reaching the same organ from a different direction has shown that carotid body glomus cells detect inflammatory lipid mediators and cytokines and adjust afferent discharge accordingly [59], and the carotid body expresses receptors for IL-1β and IL-6 in addition to TNFR1, suggesting a polymodal immune chemosensor [60].

A critical limitation of that work was its stimulus: exogenous TNF-α is a single downstream mediator rather than a physiological inflammatory challenge. This has now been addressed. In subsequent work from our group, during endotoxemia induced by lipopolysaccharide — which engages pattern-recognition receptors and triggers the full endogenous cytokine cascade — bilateral carotid body ablation attenuated the LPS-induced rise in splanchnic sympathetic nerve activity, reduced c-Fos activation of RVLM neurons, lowered the splenic noradrenaline response, and shifted the cytokine balance toward inflammation, raising TNF-α and reducing IL-10 in both plasma and spleen; splanchnic denervation produced the same shift, and more severely [61]. Three points follow. First, the carotid body behaves as a general sensor of circulating inflammatory danger signals rather than a TNF-α detector. Second, the RVLM is the sympathetic premotor node linking afferent inflammatory sensing to splanchnic efferent output, and the population it recruits is heterogeneous: only about half of the LPS-activated RVLM neurons were catecholaminergic, so inflammation engages a broader premotor architecture than the classical C1 model assumes. Third, afferent and efferent limbs are dissociable — carotid body ablation reproduced much of the cytokine phenotype of splanchnic denervation for TNF-α and IL-10, but only splanchnic denervation raised IL-6, indicating that IL-6 control depends more on local sympathetic innervation of tissues than on afferent drive. The carotid body is therefore necessary for full engagement of the splanchnic anti-inflammatory pathway, but it is not the whole of it, and the afferent organization of the reflex is best regarded as distributed and partly redundant, with vagal, nociceptor, hepatic, and chemoreceptor sensors converging on brainstem premotor circuits.

### Context-dependence and the host-defense trade-off

Whether engagement of these reflexes benefits the host depends critically on context. The carotid body experiments used exogenous systemic TNF-α, a sterile model that recapitulates the cytokine surge of sepsis rather than early localized infection, in which circulating cytokine is low and local macrophage activity is the dominant defense. In the sterile, high-cytokine setting the reflex is clearly protective: mortality in sepsis is driven by macrophage-derived cytokine storm causing organ failure rather than by insufficient macrophage activation, an inference consistent with, though not directly tested by, the cardiovascular benefit of anti-IL-1β therapy in the CANTOS trial [62]. During active infection with a replicating pathogen, however, early macrophage TNF-α is necessary for neutrophil recruitment and containment, and broad immunosuppression applied too early can impair clearance, as suggested by the stage-dependent results of cytokine-directed therapies in COVID-19 [63]. These pharmacological findings motivate, rather than test, the following model. We propose that the reflex obeys a proportionality principle analogous to the hypothalamic-pituitary-adrenal axis: at the low cytokine concentrations of contained infection the afferent signal is weak and local macrophage defense proceeds, whereas only at sepsis-range concentrations does the reflex fire strongly enough to suppress macrophage output — making it threshold-gated to engage when inflammation has become more dangerous than the pathogen.

We state this as a hypothesis because it is falsifiable, and we specify how. If the reflex is threshold-gated, graded intravenous TNF-α should recruit carotid sinus nerve discharge and splanchnic sympathetic outflow only above a defined plasma concentration, giving a sigmoidal rather than linear stimulus-response relation; carotid body denervation should leave bacterial clearance and survival intact in a low-inoculum, locally contained infection while worsening outcome in high-cytokine sterile inflammation; and the gain of the reflex should be reset, rather than merely engaged, in animals with chronically elevated baseline TNF-α. The sterile arm of this prediction now has direct support: in LPS endotoxemia, a sterile high-cytokine challenge, carotid body ablation worsens the cytokine profile rather than improving it [61]. The decisive and still untested comparison is the live-pathogen, low-inoculum arm. The model is refuted if the afferent stimulus-response relation proves linear from the lowest detectable cytokine concentrations, or if carotid body denervation impairs pathogen containment at low inoculum. These are tractable experiments in existing preparations, and we regard them as the most informative next step.

## Tissue-resident and circulating macrophage pools

The systemic reflexes of Sect.  7 are executed not in the brain but in peripheral compartments. Macrophages in the spleen, liver, gut, heart, and adipose tissue, all of which are innervated, together with blood monocytes and their bone marrow progenitors, are regulated by neural signals and produce the mediators that the nervous system detects.

### Spleen and liver

The spleen is the main site at which the nervous system controls systemic cytokine production. Its red pulp, marginal zone, CD169-expressing metallophilic, and tingible-body macrophage populations are a major source of the systemic TNF-α produced during sepsis and endotoxemia [64]. The spleen is innervated by the splenic nerve, a mainly noradrenergic sympathetic nerve reaching the organ as part of the splanchnic outflow. Consistent with the splanchnic anti-inflammatory pathway, sympathetic input restrains TNF-α production by splenic macrophages, and section of the splanchnic nerves removes this restraint [12, 53]; norepinephrine acts on β2-adrenergic receptors to elevate cAMP and suppress NF-κB activation [54], while anatomical studies find no direct projection from the vagus to the splenic sympathetic neurons [51].

The liver, also densely innervated, contains the largest tissue macrophage population, the Kupffer cells, which line the hepatic sinusoids and filter portal blood [65]. Kupffer cells express α7 nicotinic and β2-adrenergic receptors and reduce NF-κB-dependent cytokine production in response to cholinergic and noradrenergic input [1, 55, 65]. The liver also contributes to the afferent side: Niijima [66] recorded increased hepatic vagal afferent discharge in response to interleukin-1beta in the hepatoportal circulation. Because Kupffer cells are a local source of those cytokines, macrophage and afferent terminal act together — the macrophage produces the cytokine, the afferent fiber transmits the signal to the NTS — alongside the carotid body pathway of Sect. 7 [13, 66].

### Gut, heart, and adipose tissue

The gastrointestinal tract is a major site of bidirectional macrophage-neuron communication, detailed in Sects. 3 and 5 [8, 9, 35]. The heart illustrates macrophages contributing directly to the function of an electrically excitable tissue: Hulsmans et al. [67] showed that macrophages populate the atrioventricular node, form connexin-43 gap junctions with cardiomyocytes, and facilitate normal conduction, since macrophage depletion or deletion of macrophage connexin-43 produced conduction block. Cardiac macrophages express adrenergic and cholinergic receptors, and although direct evidence that autonomic input controls this coupling is lacking, the anatomy places them at the intersection of immune and neural control of the heart. In adipose tissue, sympathetic input restrains macrophage inflammatory activation while SAMs set local norepinephrine availability [9, 10], and in skin, nerve-associated macrophages support sensory nerve regeneration [33]. Synovial joints show the same arrangement with unusual clarity: the temporomandibular joint receives sympathetic innervation from the ipsilateral superior cervical ganglion, and removing it exacerbates zymosan-induced mechanical hyperalgesia while selectively raising intra-articular IL-6 — which tracks the hyperalgesia closely — without altering TNF-α [68]. Two general points emerge. The arrangement recurs across tissues: a resident macrophage population positioned alongside nerve fibers, regulated by neural mediators, and in turn shaping neuronal activity and organ function. And sympathetic control of the cytokine milieu is mediator-selective rather than global, since IL-6 in the joint depends on local sympathetic innervation just as splenic IL-6 during endotoxemia depends on splanchnic efferents rather than on carotid body afferent drive [61, 68] — an efferent, tissue-local axis running in parallel with the centrally organized reflex.

### Neural control of monocyte production, stress, and behavior

The nervous system also regulates circulating monocytes and the bone marrow progenitors that produce them. Sympathetic fibers innervate the bone marrow, and the release of hematopoietic cells into blood is under neural and circadian control: Mendez-Ferrer et al. [69] showed that progenitor release follows circadian oscillations driven by the sympathetic nervous system, in which noradrenergic signals acting on β3-adrenergic receptors on stromal cells reduce the retention chemokine CXCL12, and Nguyen et al. [70] showed that β2-adrenergic signaling and the clock gene Bmal1 control the diurnal oscillation of Ly6C-high inflammatory monocytes.

Neural regulation of the myeloid compartment is most evident under stress, and this is where the neuroimmune macrophage most directly meets behavior. Heidt et al. [71] showed that chronic variable stress raises sympathetic activity, lowers niche CXCL12, increases hematopoietic stem cell proliferation, and produces a sustained rise in circulating inflammatory leukocytes that accelerates atherosclerosis; Powell et al. [72] showed that repeated social stress increases proinflammatory gene expression in monocytes through β-adrenergic induction of myelopoiesis. Reservoir pools are mobilized by acute injury as well: Swirski et al. [73] identified a splenic reservoir of undifferentiated monocytes released to inflamed tissue, and Dutta et al. [74] showed that myocardial infarction, through sympathetic signaling to the bone marrow, accelerates inflammatory monocyte production and worsens atherosclerosis at remote sites. Critically for behavior, McKim et al. [75] showed that stress-induced, sympathetically driven monocytosis delivers inflammatory monocytes to the cerebral vasculature, where they promote neuroinflammation and anxiety-like behavior — a complete circuit from psychological stressor through sympathetic outflow and myelopoiesis to brain macrophage activation and behavioral output.

The afferent arm closes this loop, and its behavioral output is the founding paradigm of the field. Peripheral macrophage activation — the principal source of the initiating cytokines, though not demonstrably the obligatory one for any individual symptom — produces the fever, anorexia, social withdrawal, reduced exploration, and fatigue of sickness behavior — not a nonspecific malaise but a coordinated, adaptive reorganization of motivation that diverts energy toward host defense, and one that becomes maladaptive when the inflammatory signal fails to resolve [76]. The afferent and humoral routes are, however, distinct: fever depends on prostaglandin E₂ synthesized locally within the brain rather than on neural afferents, since deleting COX-2 and mPGES-1 selectively in brain endothelial cells attenuates fever while leaving inflammation-induced hypoactivity intact [77], and subdiaphragmatic vagotomy does not block intraperitoneal LPS-induced fever [78]; the behavioral components, by contrast, are signaled by vagal, hepatic, and carotid body afferents, and vagotomy blocks the LPS-induced suppression of social investigation without altering the peripheral cytokine response [79]. In humans the same circuitry is implicated in affective illness. Elevated IL-6 and C-reactive protein are associated with depression and with inflammation-associated fatigue and anhedonia, and the temporal ordering favors inflammation as antecedent rather than consequence: in a population-based birth cohort, higher serum IL-6 in childhood predicted depression and psychotic experiences in young adulthood [80]. Because the macrophage is the principal cellular source of these cytokines, and because its output is set by the autonomic tone described throughout this section, it sits upstream of the behavioral phenotype — which is why the inflammation-associated subtype of depression is now a target for immune-directed treatment, and why the reflex circuitry reviewed here is of more than physiological interest [81]. The macrophage is, in this sense, the cell through which psychological state is converted into immune state and back again.

## Innate sensing and neuroinflammation

Microglia detect pathogen- and damage-associated molecular patterns through the receptors cataloged in their sensome [22]. In the sterile environment of the CNS, damage-associated patterns dominate: ATP from injured cells acts on purinergic receptors to drive chemotaxis, while HMGB1 and aggregated proteins engage TLRs to initiate inflammatory programs [20]. This sensing is protective when it clears debris but pathological when chronic stimulation sustains a maladaptive inflammatory state.

The NLRP3 inflammasome is a central node in microglial neuroinflammation. Assembled in response to potassium efflux, reactive oxygen species, and aggregated material, NLRP3 activates caspase-1 to process IL-1β and IL-18 and to cleave gasdermin D, the effector of pyroptosis, an inflammatory form of programmed cell death increasingly implicated across neurodegenerative disease [82]. Heneka et al. [83] demonstrated that NLRP3 activation occurs in microglia in Alzheimer models and in human disease, that amyloid-β is a potent activator, and that NLRP3 or caspase-1 deficiency protects against amyloid pathology and cognitive decline — establishing inflammasome-driven IL-1β as a driver of neurodegeneration rather than a bystander.

The cyclic GMP-AMP synthase-stimulator of interferon genes (cGAS-STING) pathway has emerged as a key driver of the sterile neuroinflammation of aging and neurodegeneration. cGAS detects cytosolic double-stranded DNA, including DNA released from damaged mitochondria, and synthesizes 2’3’-cGAMP, which activates STING to drive type I interferon and NF-κB-dependent inflammation [84]. Gulen et al. [85] showed that cGAS-STING drives a senescence- and aging-associated neuroinflammatory program in microglia and that STING blockade suppresses it and ameliorates features of neurodegeneration in disease models. STING-dependent microglial activation has been implicated in Parkinson’s and Alzheimer’s disease, making the pathway a prominent therapeutic target for age-related neuroinflammation [84, 85].

## Dysregulation in disease

Microglia are now central to the pathogenesis of neurodegenerative disease. Genome-wide association studies identify microglial-expressed genes as major risk loci for Alzheimer’s disease, including TREM2, CR1, and APOE, implicating microglial phagocytosis, complement handling, and lipid metabolism [86]. Single-cell sequencing identified the disease-associated microglia (DAM) state — loss of homeostatic genes with upregulation of TREM2, APOE, CST7, and LPL — a TREM2-dependent program recruited to amyloid plaques that nonetheless fails to achieve adequate clearance [87, 88]. DAM is only one of several reactive states now resolved by single-cell and spatial profiling, so the homeostatic-versus-DAM dichotomy is itself a simplification; ontogeny adds a further axis, since disease-associated microglia and monocyte-derived disease inflammatory macrophages arise from distinct origins and can be distinguished within the same degenerating tissue, meaning that states defined by transcriptome alone may conflate cells of different lineage [89].

These states are not merely descriptive taxonomy. The TREM2-dependent program can be protective or detrimental depending on disease stage and context, and the related neurodegenerative phenotype (MGnD), driven by an APOE-TREM2 axis that suppresses the homeostatic signature, shows how a single receptor pathway can switch valence — a duality any TREM2-directed therapy must reckon with [90]. Whether discrete states or a continuum best captures microglial reactivity, and how faithfully murine states map onto human disease, remain unsettled [88], and post-translational modifications add a further layer of control over microglial inflammatory programs [91]. The ultrastructurally defined ‘dark microglia’ make the point: stress-associated, P2RY12-negative cells that expand with chronic stress, aging, and Alzheimer pathology, they are now understood as an integrated-stress-response state converging molecularly with DAM/MGnD [92].

Two pathological mechanisms recur. The first is aberrant reactivation of the developmental complement-microglia pruning program, which Hong et al. [93] linked to early synapse loss in Alzheimer models before plaque deposition, though whether microglia are strictly required remains debated. The second is cytosolic-DNA-driven cGAS-STING and NLRP3 activation, sustaining a maladaptive inflammatory state in aging microglia [83, 85]. Activated microglia also harm neurons non-cell-autonomously: microglia-derived TNF, C1q, and IL-1α drive neurotoxic reactive astrocytes that promote the death of neurons and oligodendrocytes — originally labeled ‘A1’, though the binary A1/A2 scheme has since been cautioned against in favor of multi-parameter definitions [94, 95]. In Parkinson’s disease, microglia respond to aggregated α-synuclein with inflammatory activation [96].

Microglia also retain a durable memory of prior inflammatory experience, encoded in epigenetic and metabolic states rather than antigen-specific receptors. In amyloid models, peripheral inflammatory stimulation durably reprograms microglia — acute stimulation ‘training’ and repeated stimulation ‘tolerizing’ subsequent responses — and these opposite imprints measurably alter amyloid pathology months later [97]. This trained-immunity framework reframes risk factors acting through systemic inflammation, such as infection, surgery, obesity, and aging, as agents that set the baseline reactivity of brain macrophages, and raises the possibility that part of the therapeutic window lies in the inflammatory history preceding overt neurodegeneration — a possibility for which the stress-to-myelopoiesis circuitry of Sect. 8 supplies a plausible physiological route.

The macrophage-nociceptor interface of Sect. 6 is the cellular substrate of much chronic and neuropathic pain. In inflammatory pain, the magnitude and duration of macrophage-derived sensitizing mediators determine whether transient nociceptor input resolves or progresses to chronicity [16, 44]; in neuropathic pain, persistent macrophage infiltration of injured nerves and dorsal root ganglia sustains nociceptor sensitization, prominently in diabetic and chemotherapy-induced neuropathy [15, 33, 34]. Autonomic tone sets the gain of this interface, and its loss is pro-nociceptive: sympathetic denervation of an inflamed joint amplifies both local IL-6 and mechanical hyperalgesia [68], which identifies the sympathetic-macrophage-nociceptor triad, rather than the macrophage-nociceptor pair alone, as the relevant functional unit. Because the neuroimmune interactome is injury-specific, the signaling axes relevant to different pain etiologies differ, with direct consequences for therapy [16].

Macrophages are also essential nodes of gut-brain communication. The muscularis macrophage-enteric neuron unit integrates immune status with autonomic organ function [8, 9, 35], and the dependence of microglial maturation on microbiota-derived short-chain fatty acids links the intestinal environment to the functional state of brain macrophages [41]. Sampson et al. [98] showed in a Parkinson’s model that the gut microbiota are required for microglial activation and motor deficits. In obesity, sympathetic restraint of adipose macrophages is lost, permitting the proinflammatory accumulation that drives insulin resistance, while SAM expansion increases norepinephrine clearance and blunts the sympathetic drive supporting lipolysis [9, 10] — a neuro-immuno-metabolic circuit in which macrophages and sympathetic neurons jointly determine metabolic outcome.

## Therapeutic frontiers

Because microglial survival depends on CSF1R signaling, pharmacological CSF1R inhibition depletes microglia, and withdrawal permits rapid repopulation from residual progenitors. Elmore et al. [40] established this depletion-repopulation paradigm and showed that repopulated microglia restore the homeostatic compartment. In disease models, microglial elimination or forced turnover can reduce pathology, and ‘resetting’ a dysfunctional compartment — or replacing it with healthy or engineered cells — is being actively explored [99]. Because microglial identity depends on the CNS environment, any replacement strategy must contend with instruction of the incoming cells by the diseased niche.

An alternative strategy seeks to enhance protective microglial function rather than deplete the compartment. Because loss-of-function TREM2 variants increase Alzheimer’s risk and TREM2 drives the protective DAM program, agonist anti-TREM2 antibodies have advanced into clinical testing [100]. Conversely, inhibiting maladaptive pathways — NLRP3 inhibitors, cGAS-STING antagonists, and complement inhibitors to prevent aberrant pruning — offers a parallel route to dampening the destructive arm of microglial activity while preserving its protective functions [83, 85, 93]. The challenge is to separate beneficial from harmful microglial programs, which the molecular dissection of microglial states is beginning to make feasible.

The autonomic reflexes that control macrophage cytokine production are themselves therapeutic targets. Because the efferent arm is sympathetic, engagement of β2-adrenergic signaling and modulation of splanchnic outflow are potential routes to suppress macrophage-driven inflammation [12, 52]. Vagus nerve stimulation has shown clinical benefit with reductions in TNF-α and IL-6 in rheumatoid arthritis and inflammatory bowel disease, although the efferent pathway is debated and has been attributed to downstream sympathetic outflow rather than direct vagal innervation of lymphoid organs [12, 101]. The carotid body raises the prospect of modulating the reflex at its origin — through carotid sinus nerve stimulation or pharmacological targeting of the chemoreceptor — to amplify endogenous macrophage-suppressive output in sterile inflammatory and autoimmune disease [13, 60]. As emphasized in Sect. 7, such strategies demand contextual calibration, since amplifying anti-inflammatory reflexes during active infection risks impairing host defense; the most defensible windows are sterile inflammatory conditions in which no replicating pathogen must be cleared [63].

The bidirectional macrophage-nociceptor interface offers analgesic targets distinct from conventional approaches. Specialized pro-resolving mediators reduce hyperalgesia in preclinical models, and macrophage-selective delivery of IL-10 or other polarizing signals could shift peripheral macrophages toward resolution without systemic immunosuppression [15, 50]; in neuropathic pain, interfering with chronic macrophage infiltration of injured nerves, including through CSF1R modulation, may reduce ongoing sensitization [33]. Because neuroimmune interactomes are injury-specific, analgesic strategies may need to be matched to the dominant macrophage-nociceptor axis of each pain etiology [16]. Chimeric antigen receptor macrophages [102] provide a template for engineered cells directed to clear pathological substrates, though their application to nervous system disease remains speculative.

## Conclusions and outstanding questions

The evidence reviewed here indicates that the macrophage is a constitutive component of neural tissue function rather than an immune cell that engages the nervous system only occasionally. The three organizing principles — neural instruction of identity, the macrophage as a bidirectional sensor-effector node, and the recurrence of this arc across tissues (a bona fide central reflex for the splenic anti-inflammatory circuit, and local paracrine feedback loops elsewhere) — are, in our reading, the durable conceptual core that should survive revision of any individual mechanism. Macrophage identity in nervous tissue is specified by neurons and glia; macrophages perform developmental and homeostatic functions in it; they exchange signals with neurons bidirectionally in a manner that determines the generation and resolution of pain; they act as both sensors and effectors of the autonomic reflexes regulating systemic inflammation, and thereby as the cellular substrate of sickness behavior and of inflammation-associated affective symptoms; and their dysfunction contributes to neurodegeneration, chronic pain, gut-brain disorders, and neuro-metabolic disease.

Four questions define the most productive frontiers. First, how is microglial identity established and maintained by the neural environment, and can a diseased microglial compartment be reset or replaced while ensuring that incoming cells acquire protective rather than pathological programs? Second, how can the protective and destructive functions of microglia — phagocytic clearance versus complement-driven synapse loss and inflammasome-driven neurodegeneration — be pharmacologically separated? Third, what determines the threshold and context-dependence of the autonomic anti-inflammatory reflexes, and can the afferent limbs (vagal, nociceptor, and carotid body) and the efferent splanchnic limb be engaged therapeutically without compromising host defense? Fourth, how do the injury- and tissue-specific features of macrophage-neuron interactomes translate into individualized strategies for pain, neurodegeneration, and metabolic disease?

A caveat cuts across all four. Most of the mechanisms reviewed here were established in rodents — including the autonomic reflex circuitry and the carotid body afferent — and their quantitative importance in humans is not yet established, so closing this species gap is a precondition for translation. Human-based platforms are the most direct route: iPSC-derived microglia [103], microglia-containing brain organoids [104], and xenotransplantation models in which human microglia mature within the mouse brain and adopt disease-associated states in response to amyloid pathology [105, 106]. Each carries limitations — incomplete maturation, absence of a full vascular and immune context, and the artificial chimeric niche — but together they offer a route to close the rodent-to-human gap that qualifies much of the evidence reviewed here. Where human data already exist they are encouraging: the microglial signature genes of Fig. 1 are expressed by human microglia and are downregulated once the cells leave the brain niche, direct evidence that this identity is environmentally instructed in our own species [107]; the border-associated compartments of Fig. 1 have been resolved in human tissue, with CD206/MRC1 as a pan-border marker, LYVE1 among the core signature genes, and the core signature conserved between human and mouse [108]; and the efferent reflex itself is functional in humans, where vagus nerve stimulation lowers TNF production and attenuates disease activity in rheumatoid arthritis [101]. The macrophage is, on the evidence, a subject of both neuroscience and immunology, and the traffic between the two systems is best understood as a property of one cell rather than of two fields.

## Data Availability

No datasets were generated or analysed during the current study.
